# Implementing immediate postpartum contraception: a comparative case study at 11 hospitals

**DOI:** 10.1186/s43058-021-00136-7

**Published:** 2021-04-12

**Authors:** Michelle H. Moniz, Kirsten Bonawitz, Marisa K. Wetmore, Vanessa K. Dalton, Laura J. Damschroder, Jane H. Forman, Alex F. Peahl, Michele Heisler

**Affiliations:** 1 Department of Obstetrics and Gynecology, University of Michigan, 1500 E. Medical Center Dr., Ann Arbor, MI 48109 USA; 2 Institute for Healthcare Policy and Innovation, University of Michigan, 2800 Plymouth Rd., Ann Arbor, MI 48109 USA; 3 Veterans Affairs Center for Clinical Management Research, VA Ann Arbor Healthcare System, 2215 Plymouth Rd., Ann Arbor, MI 48105 USA; 4 Department of Internal Medicine, University of Michigan, 1500 E. Medical Center Dr., Ann Arbor, MI 48109 USA

**Keywords:** Implementation, Contraception, Long-acting reversible contraception, Maternity, Perinatal, Postpartum, Qualitative

## Abstract

**Background:**

Immediate postpartum long-acting reversible contraception (LARC) is an evidence-based practice, but hospitals face significant barriers to its adoption. Our objective was to examine how organizational context (e.g., size, employee attitudes toward the clinical practice) and implementation strategies (i.e., the actions taken to routinize a clinical practice) drive successful implementation of immediate postpartum LARC services, with a goal of informing the design of future implementation interventions.

**Methods:**

We conducted a comparative case study of the implementation of inpatient postpartum contraceptive care at 11 US maternity hospitals. In 2017–2018, we conducted site visits that included semi-structured key informant interviews informed by the Consolidated Framework for Implementation Research. Qualitative measures of implementation success included stakeholder satisfaction, routinization, and sustainability of immediate postpartum LARC services. Qualitative content analysis and cross-case synthesis explored relationships among organizational context, implementation strategies, and implementation success.

**Results:**

We completed semi-structured interviews with 78 clinicians, nurses, residents, pharmacy and revenue cycle staff, and hospital administrators. Successful implementation required three essential conditions: effective implementation champions, an enabling financial environment, and hospital administrator engagement. Six other contextual conditions were influential: trust and effective communication, alignment with stakeholders’ professional values, perception of meeting patients’ needs, robust learning climate, compatibility with workflow, and positive attitudes and adequate knowledge about the clinical practice. On average, sites used 18 (range 11-22) strategies. Strategies to optimize the financial environment and train clinicians and staff were commonly used. Strategies to plan and evaluate implementation and to engage patients emerged as promising to address barriers to practice change, yet were often underused.

**Conclusions:**

Implementation efforts in maternity settings may be more successful if they select strategies to optimize local conditions for success. Our findings elucidate key contextual conditions to target and provide a menu of promising implementation strategies for incorporating recommended contraceptive services into routine maternity practice. Additional prospective research should evaluate whether these strategies effectively optimize local conditions for successful implementation in a variety of settings.

**Supplementary Information:**

The online version contains supplementary material available at 10.1186/s43058-021-00136-7.

Contributions to the literature

- The literature on implementation efforts in maternity settings is quite sparse.
- Our work newly evaluates how organizational context and implementation strategies affect efforts to implement evidence-based contraceptive services in maternity settings.
- Our findings advance the literature by identifying key contextual conditions to target and providing a menu of promising strategies to include in multicomponent interventions to implement recommended contraceptive services in maternity settings.
- Moreover, our findings advance the science more broadly by beginning to illuminate mechanisms for successful clinical practice change in maternity settings, suggesting that implementation efforts should consider local context and select strategies to optimize conditions for success.

## Background

Immediate postpartum long-acting reversible contraception (LARC)—the insertion of an intrauterine device or contraceptive implant during the delivery hospitalization—is one safe and effective evidence-based intervention for family planning after childbirth [1–4]. Many women express interest in and utilize inpatient LARC services when they are available [5–7]. However, although national guidelines recommend universal access to this service, it is provided almost exclusively at a small number of “early adopter” academic medical centers [8, 9]. Utilization rates in national samples across the USA remain low (<1%) [9, 10]. Provision of immediate postpartum LARC is now reported in the Centers for Medicare and Medicaid Services’ Core Measure set [11], and many states and perinatal quality collaboratives are working to improve access to immediate postpartum LARC for interested individuals [12–18].

Hospitals, however, face significant barriers to offering inpatient LARC services. Non-reimbursement has historically impeded service provision. As insurance payment has become increasingly common, more hospitals have tried to launch these services, but with mixed success [8, 13, 14, 17, 18]. It is unclear why some hospitals succeed, while others do not. Prior work on clinical practice change suggests that both organizational context and implementation strategies are important [19, 20]. *Organizational context* refers to all the characteristics of an organization that are not part of the clinical practice itself, such as size, interconnectedness of employees, and employee attitudes toward the clinical practice. *Implementation strategies* refer to the actions taken to optimize context for change and routinize a new clinical practice. Better understanding context and strategies relevant for implementing evidence-based interventions for peripartum contraceptive services could help improve provision of this care and more broadly inform the design of maternity practice change interventions.

As a case example for better understanding implementation processes in maternity settings and to help inform the design of future implementation interventions, we examined how context and strategies drove successful implementation of immediate postpartum LARC services in early adopter hospitals.

## Methods

We conducted a comparative, multiple case study in early adopter hospitals in the USA. We employed this design with a goal of analyzing similarities and differences across cases in order to produce generalizable knowledge about how and under what circumstances implementation unfolds successfully [21, 22]. We report our methods according to the Consolidated Criteria for Reporting Qualitative Research (COREQ) [23]. We selected COREQ because of its detailed focus on the collection, analysis, and reporting of interview data, such as that used in the current study. The completed checklist is available in Additional file 1.

Because LARC service provision at the hospital level is difficult to identify within national administrative datasets, we conducted a systematic literature search in PubMed to find published literature related to research studies on immediate postpartum contraceptive care. Seventeen unique academic medical centers were identified. Study authors were contacted by email to assess site eligibility; six sites were excluded (two did not respond, and four only offered immediate postpartum LARC in the context of a research study). For each hospital, we first identified the “champion” (i.e., the individual leading implementation efforts) and invited them by email to participate in an initial telephone interview for a research project studying implementation of evidence-based peripartum contraceptive care [24–26]. We asked about their experiences with implementation, including potential organization and patient population characteristics that might have impeded or promoted implementation. Two sites declined further participation. Snowball sampling with the remaining nine sites recruited two additional hospitals that had recently implemented services and had not previously conducted research trials of peripartum contraceptive care.

Between August 2017 and September 2018, we conducted single-day site visits, which included semi-structured interviews with key informants (i.e., individuals identified by the champion as having unique knowledge about implementation based on their role within the organization), with a goal of representing various stakeholder groups’ perspectives in describing implementation (e.g., clinicians, pharmacists, revenue cycle staff, hospital administrators) [24, 25, 27]. Interviews were conducted by MHM and MKW and were audio-recorded with permission and professionally transcribed verbatim. Rarely, due to key informants’ availability, interviews were completed by telephone (*n*=4) or email (*n*=1). Field notes were taken during interviews and used to develop memos reviewed during analysis.

### Theoretical framework

The Consolidated Framework for Implementation Research (CFIR) guided this research a priori, informing data collection (i.e., semi-structured interview guide) and analyses (i.e., coding framework). CFIR includes 39 contextual conditions that may influence implementation of an evidence-based practice [28]. Our interview guide and analysis were also informed by the Expert Recommendations for Implementing Change (ERIC)—an evidence-based list of 73 discrete strategies that can be bundled in a multicomponent intervention for implementation—which was used to characterize the actions taken at each site to optimize conditions for implementation and routinize inpatient LARC services [20]. The interview guide contained items and probes about each CFIR construct. We also included probes about specific ERIC strategies. Specific items and probes in the interview guide were refined via pilot testing with our institution’s interdisciplinary Program on Women’s Healthcare Effectiveness Research (PWHER). PWHER members include academic women’s health clinicians and health services researchers, many with specific expertise in contraceptive care delivery. Group members provided feedback about CFIR constructs and strategies thought to be less likely relevant to postpartum contraceptive care, guiding the authors’ development of a more parsimonious interview guide. The codebook retained all CFIR constructs and ERIC strategies, to allow all potentially relevant themes and relationships to emerge from the data during analysis.

Of note, “champions” appear as a condition in CFIR (“formal implementation leader”) and a strategy in ERIC (“identify and prepare champions”); moreover, as implementation leaders, champions are often the source of other ERIC strategies. Because of their central role in implementation, we focus on champions as a condition, but also describe the implementation strategies they executed.

### Analysis

**Fig. 1:**
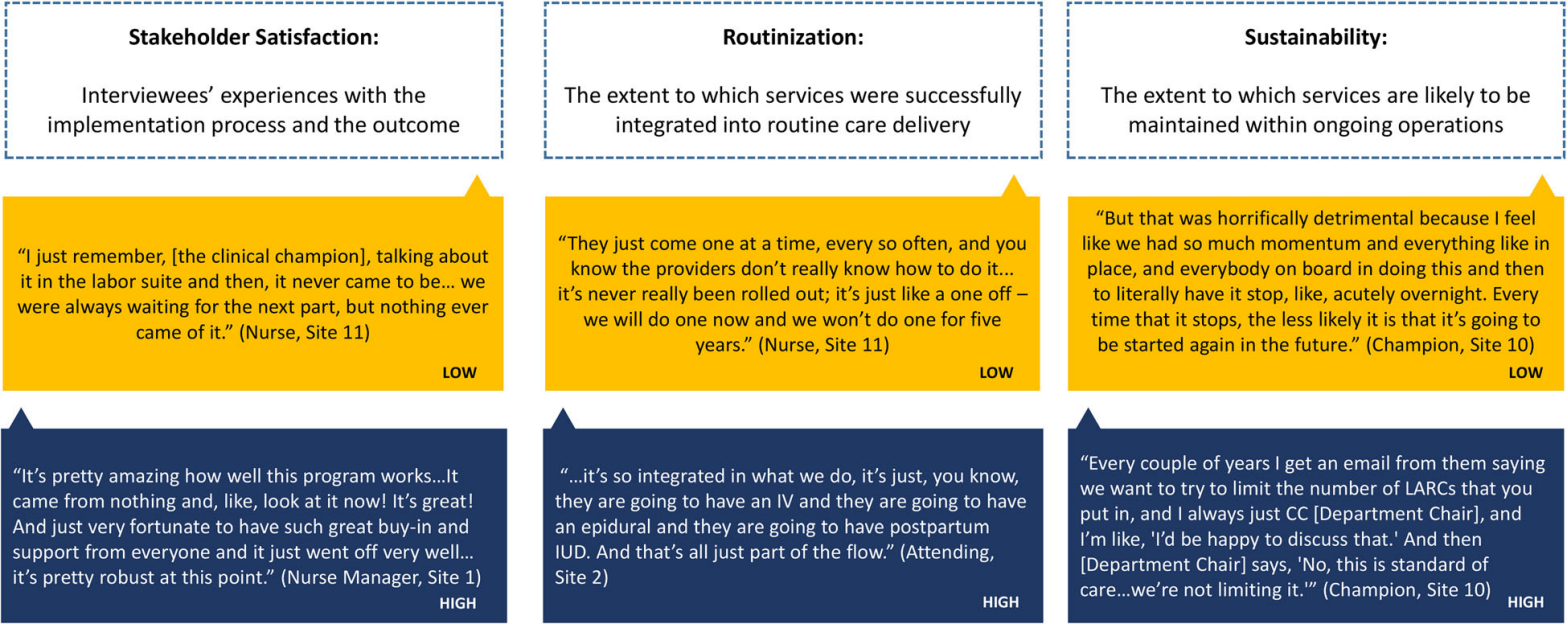
Qualitative definitions of implementation success

Consensus coding was used throughout, with MHM, KB, and MKW leading the coding process using Dedoose software. In order to understand the relationship between organizational context, strategies, and implementation outcomes, we first devised a three-part qualitative definition of implementation success, based on (1) stakeholder satisfaction, (2) routinization, and (3) sustainability (Fig. 1) [29]. These three outcomes were selected based on their amenability to qualitative measurement and observed variation across sites; other outcomes more suitable to quantitative measurement (e.g., implementation costs, adoption, fidelity) or without variation across sites (e.g., feasibility was universally low) were not included. We assigned ratings of low, medium, or high for each component of implementation success at each site. Second, we conducted a qualitative content analysis, using CFIR constructs as a priori codes, to understand the local conditions for implementation. For each site, coded data were then assigned quantitative ratings indicating valence and strength of influence (−2 [strong barrier] to +2 [strong facilitator]) of each CFIR condition on implementation success, as previously described [30] and using the criteria in Table A.1. We summed these ratings in a contextual summary score to understand the relative prevalence of positive and negative influences at each site. Third, we identified implementation strategies used by each site, using the ERIC strategies as an initial checklist and allowing additional strategies to emerge from the data. Finally, we developed matrices with CFIR conditions and associated strategies as rows and hospital cases in columns to support cross-case synthesis investigating (1) conditions most strongly associated with implementation success, including conditions that are *essential* (e.g., necessary) versus *highly influential* (e.g., catalyzing or inhibiting) for implementation success and (2) promising strategies to promote implementation success, based on those effectively used by sites or emerging as underused (i.e., potentially helpful to mitigate unaddressed barriers).

Findings were member-checked with site implementation leaders by phone. Our research team was all female, including research assistants (KB, MKW) and physicians with training in qualitative research (MHM, VKD, MH, AFP) and obstetrics and gynecology (MHM, VKD, AFP). Consultation on qualitative research was provided by researchers with extensive experience in the field and deep familiarity with the implementation literature (LJD, JHF).

## Results

We interviewed 78 key informants (average key informants per site, 7.1 [range 5–10]; mean interview duration, 35 min [range 11–65]) in 11 maternity hospitals (Table 1). Nine sites participated in member-checking phone calls.

**Table 1:** Interviewee characteristics

Interviewees	***N***=78
Frontline clinicians	45
Implementation leaders^a^	12
Other attending physicians	12
Residents	9
Nurses	9
Midwives	3
Operations Staff	24
Pharmacy staff	10
Administration	4
Revenue cycle staff	7
Project managers	3
Hospital Leadership	9

^a^All attending physicians

Implementation success varied across sites, with site 11 notably unsuccessful across all domains and site 10 implementing services, but with low sustainability prompting de-implementation (Table 2). We identified nine contextual factors as essential or highly influential conditions for successful implementation. The contextual summary scores suggested that some sites enjoyed a highly enabling context for implementation, while others faced more mixed or unfavorable conditions. On average, sites used 18 (range 11–22) implementation strategies (Table 3).

**Table 2:** Relationship between site context, qualitative themes, and implementation success

Characteristic	Site 1	Site 2	Site 3	Site 4	Site 5	Site 6^a^	Site 7	Site 8	Site 9	Site 10	Site 11^a^
Organizational characteristics											
Annual delivery volume	5500	3000	2400	3500	3000	5500	3000	2500	4500	4000	8500
Number of attendings on labor and delivery	36	30	15	33	45	NA^b^	77	20	51	50	50
Qualitative measures of implementation success											
Stakeholder satisfaction	High	High	High	High	Med	Med	Med	Med	Med	High	Low
Routinization	High	High	High	High	High	High	High	High	High	High	Low
Sustainability	High	High	Med	Med	Med	Med	Med	High	High	Low	NA^b^
Organizational context for implementation^c^											
*Essential conditions*											
Implementation champion(s)	2^**+**^	2^**+**^	2^**+**^	2^**+**^	2^**+**^	2^**+**^	2^**+**^	1^**+**^	1^**+**^	2^**+**^	2^**-**^
Financial environment	2^**+**^	2^**-**^	1^**+**^	2^**-**^	2^**-**^	1^**-**^	2^**-**^	2^**-**^	0	2^**-**^	2^**+**^
Hospital administrators’ engagement	2^**+**^	2^**+**^	1^**+**^	2^**+**^	1^**+**^	1^**+**^	1^**X**^	2^**+**^	1^**+**^	1^**+**^	2^**-**^
*Highly influential conditions*											
Networks and communications	2^**+**^	2^**+**^	2^**+**^	1^**X**^	0	2^**X**^	1^**+**^	1^**+**^	0	1^**X**^	1^**X**^
Compatibility with norms and values	2^**+**^	2^**+**^	2^**+**^	1^**+**^	2^**+**^	2^**+**^	1^**+**^	2^**+**^	1^**+**^	2^**+**^	1^**+**^
Patient needs and resources	2^**+**^	2^**+**^	2^**+**^	1^**+**^	2^**+**^	2^**+**^	1^**+**^	2^**+**^	1^**+**^	2^**+**^	2^**X**^
Learning climate	2^**+**^	1^**+**^	2^**+**^	2^**+**^	2^**+**^	2^**+**^	1^**+**^	1^**X**^	0	2^**+**^	2^**X**^
Compatibility with workflow	1^**X**^	1^**-**^	1^**+**^	0	1^**-**^	1^**-**^	1^**+**^	1^**-**^	1^**-**^	0	2^**X**^
Clinician/staff attitudes, beliefs, & knowledge	2^**+**^	2^**+**^	2^**+**^	2^**+**^	1^**+**^	1^**+**^	2^**X**^	2^**X**^	2^**X**^	1^**+**^	1^**-**^
*Contextual summary score*^d^	34	32	34	27	16	19	10	1	2	17	-18
Overview of implementation process											
Number of implementation strategies used	20	16	22	19	17	16	20	22	11	16	13
Duration of implementation (months)	16	36	4	7	48	18	60	10	12	27	NA^e^

^a^Member-checking was not completed for these sites

^b^Data unavailable

^c^Organizational context ratings reflect influence of the contextual factor on implementation at each site, 2^+^ denotes a strong positive influence, 1^+^ denotes a weak positive influence, 1^-^ denotes a weak negative influence, 2^-^ denotes a strong negative influence, 2^X^ denotes a strong mixed influence, 1^X^ denotes a weak mixed influence, 0 denotes no apparent influence

^d^Summary score reflects the sum of ratings for all 39 CFIR constructs (Score = [positive] – [negative + mixed])

^e^Implementation was ongoing at time of interview

**Table 3:** Implementation strategies for immediate postpartum contraceptive services and frequency of utilization by study sites

Strategies^a^	Sites utilizing strategy											
	Total	1	2	3	4	5	6	7	8	9	10	11
Strategies to plan and lead implementation												
*Identify and prepare champion(s):* identify and prepare individuals who dedicate themselves to supporting a new practice and overcoming indifference or resistance	11	■	■	■	■	■	■	■	■	■	■	■
*Build an implementation coalition*: recruit and cultivate relationships with partners in the implementation effort	10	■	■	■	■	■	■	■	■		■	■
*Promote network weaving:* cultivate high-quality working relationships within and across organizational units to promote information sharing, collaborative problem-solving, and a shared vision related to implementing the innovation	9	■	■	■	■	■	■	■	■		■	
*Conduct local consensus discussions:* include stakeholders in discussions about whether the clinical innovation appropriately addresses an important problem	5			■			■		■		■	■
*Conduct local needs assessment*: collect and analyze data (e.g., baseline contraceptive counseling and use rates) related to the need for the innovation	3			■					■	■		
*Assess for readiness; identify barriers and facilitators*: assess various aspects of an organization to determine its readiness to implement, barriers that may impede implementation, and strengths that can be used in the implementation effort	0											
*Tailor strategies*: tailor the implementation strategies to address barriers and leverage facilitators that were identified through earlier data collection	0											
*Develop a formal or informal implementation blueprint*: Develop a description of the (1) aim/purpose of the implementation, (2) scope of the change (e.g., units affected), (3) timeframe and milestones, and (4) appropriate performance measures	4	■		■	■			■				
*Obtain stakeholder feedback about the implementation plan*: formally and informally soliciting front-line workers’ opinions to refine the implementation plan	2							■	■			
*Facilitation:* a process of interactive problem-solving and support in the context of a recognized need for improvement and a supportive interpersonal relationship	9	■	■	■	■	■	■	■	■		■	
*Assess and redefine workflow*: map current work processes and plan for desired work processes, identifying changes necessary to routinize the clinical innovation	5	■		■	■			■			■	
*Stage implementation scale up*: phase implementation efforts by starting with small pilots or demonstration projects and gradually move to a system-wide rollout	10	■	■	■		■	■	■	■	■	■	■
Strategies to optimize financial environment												
*Access new funding*: access money to facilitate implementation	11	■	■	■	■	■	■	■	■	■	■	■
*Place innovation on FFS lists/inpatient formulary*: work to place the clinical innovation on lists of actions for which providers can be reimbursed (e.g., a drug is placed on a formulary, a procedure is now reimbursable)	11	■	■	■	■	■	■	■	■	■	■	■
Strategies to optimize for infrastructure change												
*Change record systems*: change electronic medical records to allow better patient care or assessment of clinical outcomes	10	■	■	■	■	■	■	■	■		■	■
*Change physical structure and equipment*: adapt the physical structure/equipment to accommodate the intervention (e.g., adding a Pyxis^TM^ or device insertion supplies)	11	■	■	■	■	■	■	■	■	■	■	■
Strategies to train, educate, and support clinicians and staff												
*Provide dynamic training and educational activities:* use interactive methods to teach stakeholders (e.g., providers, operations staff) about the innovation	11	■	■	■	■	■	■	■	■	■	■	■
*Develop and distribute educational materials*: disseminate manuals and toolkits	10	■	■	■	■	■		■	■	■	■	■
*Conduct ongoing training*: offer follow-up training, advanced training, booster training, purposefully spaced training, training to competence, structured supervision	10	■	■	■	■	■	■	■	■	■	■	
*Remind clinicians:* develop reminder systems designed to help clinicians to recall information and/or prompt them to use the clinical innovation	10	■	■	■	■	■	■	■	■	■	■	
*Provide clinical supervision*: expert clinician offers ongoing supervision	4	■			■				■			■
*Organize clinician and staff team meetings*: support the teams implementing the innovation and protect time to reflect on their efforts and share lessons learned	5	■		■	■		■		■			
*Engage local opinion leaders*: activate individuals identified by colleagues as “influential” to motivate colleagues to adopt the clinical innovation; dampen resistance among opinion leaders, if needed	0											
Strategies to engage patients												
*Prepare patients to be active participants*: prepare patients to inquire about care guidelines and available treatment options and request the clinical innovation from their providers desired	6	■				■		■	■	■		■
*Involve patients in implementation planning*: solicit and use patient feedback	0											
*Engage community resources*: utilize health departments, non-profits, resources for addressing social determinants of health, and reproductive justice experts	1		■									
Strategies to evaluate Implementation												
*Plan for outcome evaluation*: identify relevant outcomes, measures, and data sources	2			■				■				
*Develop processes and tools for quality monitoring*: develop, test, and utilize systems and procedures to monitor clinical processes or outcomes related to the innovation	5			■	■	■			■		■	
*Evaluate the implementation*: monitor progress and adjust clinical practices and implementation strategies to continuously improve the quality of care	8	■	■	■	■	■	■	■	■			
*Audit and feedback*: collect clinical performance data and give it to clinicians and administrators to monitor, evaluate, and modify provider behavior	0											

^a^Adapted from the Expert Recommendations for Implementing Change (Powell, et al., *Implementation Science,* 2015)

NOTE: Strategies used by sites but not clearly linked to implementation outcomes include: conduct cyclical small tests of change (*n*=1), use advisory boards and work groups (*n*=2), use train the trainer strategies (*n*=1), and revise professional roles (*n*=1)

*LARC* Long-acting reversible contraception

**Fig. 2:**
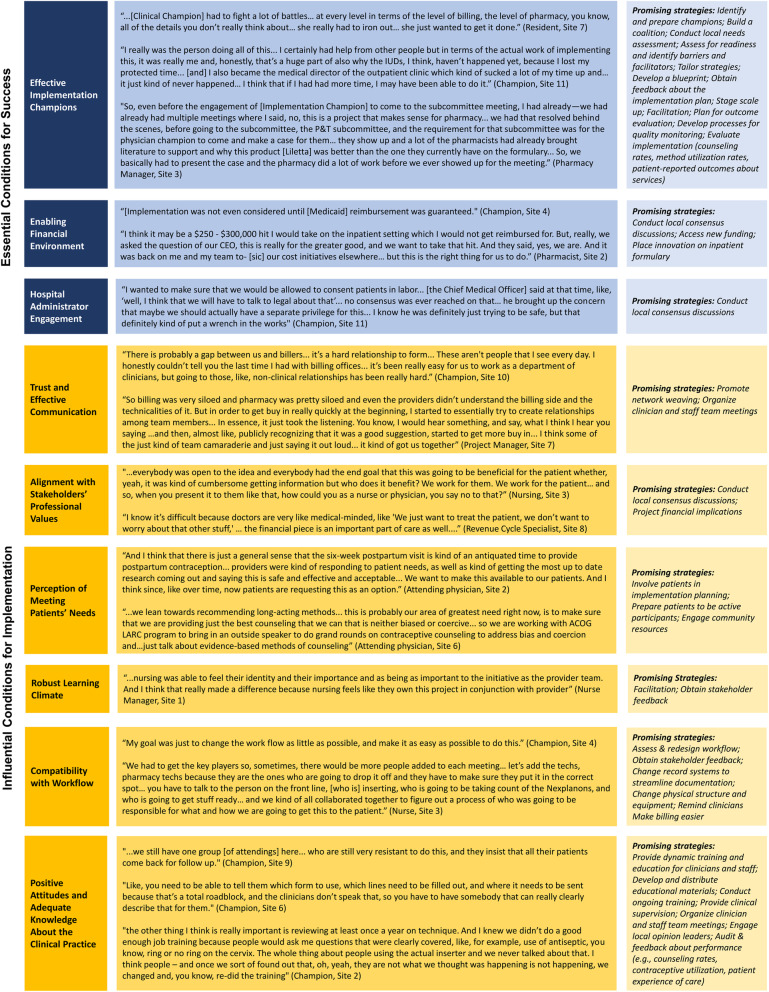
Qualitative data illustrating the effects of contextual conditions on implementation

Implementation success was driven by complex interactions between context and implementation strategies. We use qualitative data to illustrate how each condition influenced implementation and describe promising strategies for optimizing each condition for implementation success (Fig. 2).

### Essential conditions for success

#### Effective implementation champions

##### Condition

Implementation champions (i.e., the clinician(s) leading implementation and the team they built) were crucial for success. All clinical champions were obstetrician-gynecologists (*n*=5 generalist, *n*=6 family planning, *n*=1 maternal-fetal medicine). Effective clinical champions were described as trusted by colleagues, demonstrating grit and resilience, and using a participatory leadership style. Site 11’s failure to launch services was partly related to an ineffective clinical champion, who described competing demands on her time and feeling overwhelmed by challenges encountered. Clinical champions often required support from an interprofessional team, whose members addressed barriers in organizational silos outside the clinical champion’s reach. For example, one site’s pharmacy manager described how he dissipated resistance from colleagues before the clinical champion ever formally introduced the initiative. Clinical champions and teams effected change at many levels—advocating for more favorable payer reimbursement policies, engaging hospital administrators, driving operations and infrastructure changes, and building support for change among their colleagues.

##### Strategies

Though all sites had an identified clinical champion, none had any training or tools to prepare them for their highly influential role in implementation. Nearly all cited need for more support (e.g., protected time, administrative support). Two sites used project managers, who reduced burden on the clinical champion and offered a unique skillset for facilitating teamwork and problem-solving. Clinical champions and teams often cited the utility of scale-up approaches (e.g., initially launching one LARC device type, piloting services with a small group of providers). Few champions/teams utilized implementation planning strategies, and many experienced ensuing inefficiencies, need for adaptations, and stakeholder frustration. No implementation teams robustly evaluated implementation outcomes (e.g., by monitoring implementation costs, stakeholder satisfaction, adoption of new workflows, patient utilization of immediate postpartum LARC, or the patient experience of postpartum contraceptive care).

#### An enabling financial environment

##### Condition

Payer reimbursement policies profoundly affected implementation success. All hospitals faced risk of financial losses associated with providing inpatient LARC services due to non-universal reimbursement by payers. Evidence of financial losses often led to interruptions in service provision and, at site 10, program de-implementation.

##### Strategies

Many sites described advocating for public and private payer reimbursement for inpatient LARC care. Some hospitals used private donor-sponsored LARC devices to minimize financial losses and thereby expand access to services, but grants were time-limited, and patient demand could outpace supply. Some implementation teams successfully used consensus discussions to increase hospital administrators’ willingness to absorb financial losses.

#### Hospital administrator engagement

##### Condition

Opposition from hospital administrators nearly guaranteed implementation failure. Site 11 interviewees described how implementation was encumbered by department leaders’ disagreement with the clinical champion about processes for training providers and consenting patients for inpatient intrauterine devices (IUDs), and ultimately, lack of permission to proceed with implementation. Some other sites’ leaders were “philosophically onboard,” but grappled with the potential financial implications of offering services. If supportive, hospital administrators could promote sustainability of service provision.

##### Strategies

Local consensus discussions were important to overcome opposition and secure buy-in from administrators.

### Highly influential conditions for success

#### Trust and effective communication

##### Condition

Many sites lacked pre-existing relationships and communication processes across clinicians and operations staff and struggled to build trust among individuals with divergent expertise, priorities, and reporting structures. Mistrust and ineffective communication undermined the collaboration necessary to address implementation challenges and ensure efficient frontlines care delivery.

##### Strategies

Network weaving (i.e., intentional efforts to cultivate high-quality working relationships) and creating infrastructure for shared problem-solving and accountability (e.g., recurring team meetings with a shared implementation task list) were helpful strategies to support the implementation team. Many champions used standing meetings among divisions and unit committees to facilitate dialogue and strengthen relationships across otherwise siloed frontlines clinicians and staff.

#### Alignment with stakeholders’ professional values

##### Condition

Successful implementation relied on stakeholders believing the new practice aligned with their professional values. Clinicians generally embraced the new practice, driven by perceptions that enhancing contraceptive access was central to their professional mission. Conversely, pharmacists and billing specialists often described inpatient LARC provision as a “money-losing proposition” at odds with their fiduciary obligations. Clinician frustration that the “device gatekeepers” were not won over by evidence of the initiative meeting patients’ needs and operations staff wariness that clinicians would distribute devices without genuine consideration of the hospital’s financial sustainability often jeopardized implementation. Site 6 spent 4 years addressing this barrier.

##### Strategies

Occasionally, champions effectively engaged operations staff by using consensus discussions to align the initiative with the hospital’s clinical mission. Usually, more pragmatic approaches were needed (e.g., projecting potential financial outcomes, providing proof of payment for a handful of pilot test devices).

#### Perception of meeting patients’ needs

##### Condition

Many interviewees characterized immediate postpartum LARC services as an important opportunity to address unmet patient needs related to access barriers and individuals’ preferences for contraceptive care (e.g., IUD insertion under regional anesthesia). In most sites, perceptions that offering inpatient LARC services better met patients’ needs strongly promoted adoption and stakeholder satisfaction. Many interviewees described tensions between enhancing contraceptive access while also promoting patient-centeredness and equity of care.

##### Strategies

No sites involved patients or patient advocacy groups in implementation planning or evaluation. Most sites engaged in some efforts to prepare patients to be active participants in care (e.g., developing educational handouts about contraceptive options).

#### Robust learning climate

##### Condition

Robust learning climates, where clinicians and staff described feeling essential and empowered to shape change, catalyzed implementation and stakeholder satisfaction. In strong learning climates, champions could actively partner with colleagues to design new clinical workflows, problem-solve around challenges, and make real-time refinements to care delivery post-implementation.

##### Strategies

Champions promoted a positive learning climate by engaging in facilitation (a process of interactive problem-solving), expressing curiosity about colleagues’ needs and involving them in decision-making, creating psychological safety for clinicians trying a new practice (e.g., giving their cellphone number to call whenever needed), and making colleagues’ contributions to implementation visible to peers and leaders.

#### Compatibility with workflow

##### Condition

Embedding inpatient LARC into daily care delivery routines required steps to minimize workflow disruptions, including establishing communication processes across teams and settings, making devices readily available, optimizing the electronic medical record for documentation and device ordering, and streamlining billing and coding processes. At most sites, workflow changes developed organically, often resulting in inefficiencies, provider confusion and frustration, interruptions to service provision, need for adaptations, and dampening of stakeholder satisfaction.

##### Strategies

At two sites, implementation teams effectively improved workflow compatibility by prospectively involving everyone affected by changes and, at one site, using a workflow process map to delineate roles and responsibilities. Multiple sites changed electronic medical records (e.g., creating a standardized documentation element for contraceptive counseling) and purchased new equipment (e.g., long forceps for IUD insertion).

#### Positive attitudes and adequate knowledge about the clinical practice

##### Condition

All sites described how individual clinicians with negative perceptions could insidiously undermine service delivery. Nurses concerned about adverse effects on breastfeeding or providers worried about expulsion rates could preclude some patients from meaningful access, even when inpatient LARC services were “available” at a site. Clinicians and staff also had significant knowledge and skill gaps regarding immediate postpartum LARC.

##### Strategies

Champions and team members were crucial for overcoming resistance and addressing informational needs. Most champions led dynamic trainings, including didactics (e.g., Grand Rounds, training sessions for billing staff) and hands-on simulation training in postplacental IUD insertion. One-time training was often described as insufficient; champions cited need for ongoing training and clinical supervision. Strategies that might have helped address individuals’ resistance to change include meetings to reflect on the new practice and share lessons learned, activating local opinion leaders, and providing performance audit and feedback.

## Discussion

We identify key contextual conditions to target and a menu of promising strategies to inform the design of future multi-component interventions for implementing immediate postpartum LARC care.

Our findings suggest that immediate postpartum LARC implementation should focus first on supporting champions, creating an enabling financial environment, and engaging hospital leaders. Prior work has emphasized the importance of champions for immediate postpartum LARC implementation [8, 13, 17, 31]. The current study sheds light on why and how champions are so essential, by newly identifying the myriad strategies champions deploy to promote successful implementation. Our results suggest that many champions need more support, including protected professional effort and administrative support from project managers, as other research notes [32]. Our findings also call for multidisciplinary implementation teams and suggest that efforts may benefit from including trained implementation scientists. A recent integrative review identified only five studies of solo vs. team champions for clinical practice change efforts; all documented that teams benefit from initiatives requiring complex behavior change [33–37]. We add rich qualitative evidence that implementation teams are essential for boundary-spanning—helping champions address barriers in organizational silos outside their sphere of influence [38]. Specifically, we found that implementation team members helped champions identify barriers, mitigate emerging challenges, and act as opinion leaders to engage their peers. These findings have important implications for the design, implementation, and evaluation of implementation interventions, suggesting that the work of measuring barriers and facilitators, selecting aligned implementation strategies, deploying strategies, and evaluating and refining implementation efforts may best be achieved by implementation teams that maximally leverage members’ professional relationships and knowledge of local culture. An enabling financial environment was also essential to implementation success, as others have noted [17, 39–41]. Efforts to promote universal reimbursement and seamless payment processes for inpatient LARC services would remove a major obstacle to hospital adoption of this care and promote more equitable patient access. Additionally, we newly document how hospitals may engage in creative strategies to mitigate or absorb potential financial losses, even in the current coverage climate.

We identified multiple inefficiencies in implementation, suggesting the need for additional implementation strategies. Sites may have benefited from more intentional efforts to plan implementation, build relationships across organizational silos, dampen resistance from individual clinicians, and evaluate quality of care outcomes (e.g., contraceptive counseling rates, contraceptive utilization, patient experience of care). These findings may seem obvious, but this study suggests that these efforts are not currently being undertaken. Such efforts may have more efficiently and effectively addressed barriers related to networks and communications, compatibility with norms and workflow, and individual clinicians’ resistance to change and guided efforts to optimize the efficiency, equity, and patient-centeredness of care delivery processes.

The paucity of efforts to engage patients in implementation surprised us. At all sites, clinicians cited a desire to better meet patient needs as the impetus for launching inpatient LARC services, but did not invite patients to participate in the design or evaluation of new services. Meaningfully engaging patients in redesigning healthcare services is associated with improved outcomes and healthcare quality [42]. Patient engagement may be particularly important for contraceptive services. Historically and in contemporary practice, women of color, immigrants, incarcerated individuals, youth, individuals with disabilities, and lower income people have experienced being directed or coerced into using particular contraceptive methods [43–45]. It is thus crucial that efforts to improve contraceptive care quality include patients and communities in program design and evaluation, with an explicit goal of promoting patient-centeredness and reproductive justice.

At all sites, successful maternity practice change was complex, requiring significant human capital and coordination across diverse stakeholders. Though implementation is often framed as complex across many sectors of healthcare, maternity care may be exceptionally so. Butler et al [46] refer to complexity with respect to intervention, multiple synergies, multiple professions involved, the need for adaptations to ensure strong fit between existing and new processes, and engagement by diverse professions and roles across multiple organizational boundaries. Maternity care is exceptionally complex: inpatient maternity units provide emergency services for pregnant individuals, host operating rooms and sometimes intensive care beds, and deliver routine labor, delivery, and postpartum recovery services for mothers and infants. Volume and acuity of care are often unpredictable. The importance of workflow compatibility is particularly important to the maternity setting and must include multiple services and departments. Our findings, however, highlight a relative lack of pre-existing relationships across the many healthcare workers who contribute to care delivery (e.g., clinicians, billing staff, pharmacy staff). There are increasing calls for more meaningful involvement of patients in process redesign [47–49]. In our study, use of postpartum contraception, the clinical innovation, is largely driven by patient preferences; thus, user-centered design approaches may be particularly important for uptake. While it is not necessarily surprising that planning and evaluating implementation efforts are important, we, like others, have found that these activities are inconsistently done. Our findings call for more support for individuals leading change in complex care settings, to enable robust implementation planning and evaluation activities and more active approaches to engaging patients in redesign of maternity services.

A key working assumption within implementation science is the need to tailor implementation strategies to address contextual barriers and leverage facilitators [50, 51]. Researchers have highlighted the importance of understanding contextual influences on implementation and then carefully choosing strategies and designing parameters of those strategies based on knowledge of context [50–52]. Our findings provide a mapping from contextual determinants using a widely cited implementation science framework and mapping actual use of strategies to the ERIC list of strategies, also widely cited. Others have identified combinations of strategies leading to better outcomes [53, 54] or using a group process to choose strategies to use in future phases of work [55]. Our study is unique in that first, we describe a process to identify high-priority contextual factors that appear to be associated with desired outcomes; then, based on those determinants, we describe promising strategies used by higher-performing hospitals or that were underused and had potential for addressing each high-priority determinant. Qualitative data provide rich detail about how determinants manifest within the inpatient maternal settings and how strategies relate to those determinants. Strategies paired to determinants largely align with recommendations from the CFIR-ERIC Matching Tool [52]. Thus, our findings provide an initial set of strategies based on implementation experiences across 11 hospitals providing inpatient postpartum LARC services, and at the same time, provide support for earlier work to match ERIC strategies to address contextual barriers. An important step for future work will be to further operationalize [50, 56] and prospectively evaluate these strategies in a prospective, multi-site trial conducted in heterogeneous maternity care settings. Such research will further illuminate mechanisms for successful clinical practice change in complex care settings.

Study strengths include rich contextual variation across sites, results achieved in real-world settings, and robust qualitative methodology. Our study design is also subject to limitations. Our sampling strategy identified only academic, early adopter hospitals. Most immediate postpartum LARC is provided at academic centers [8, 9], so this was a reasonable sample. Specific contextual factors and strategies identified here may not be generalizable to all settings, but our findings underscoring the importance of intentionally designing implementation interventions to address local context are presumably applicable across settings. Cross-sectional interviews are subject to recall and social desirability bias and may not have captured subtle attitudes or changes in context or outcomes. We did not include patients in this study, due to our focus on implementation and feasibility at the hospital level. This is a notable limitation. Our findings emphasize the critical need to evaluate patients’ preferences and experience of care to better guide efforts to improve peripartum contraceptive services.

## Conclusions

Implementation efforts in maternity settings should consider local context and select strategies to optimize conditions for success. Our findings provide a roadmap for this process, elucidating the key contextual conditions to target and providing a menu of promising implementation strategies for embedding recommended peripartum contraceptive care into routine maternity practice.

## Supplementary Information

**Additional file 1.** COREQ checklist. PDF file COREQ (COnsolidated criteria for REporting Qualitative research) Checklist Items included in report and respective page numbers on which they appear

**Additional file 2. Table A.1** Criteria^1^ for assigning quantitative ratings to CFIR constructs.

## Data Availability

The datasets used and/or analyzed during the current study are available from the corresponding author on reasonable request.

## Abbreviations

LARC: Long-acting reversible contraception
CFIR: Consolidated Framework for Implementation Research
ERIC: Expert Recommendations for Implementing Change
IUD: Intrauterine device
