# Supplementary material for: Implementing immediate postpartum contraception: a comparative case study at 11 hospitals
Source: Implement Sci Commun. 2021 Apr 12;2:42. doi: 10.1186/s43058-021-00136-7 (PMC8042857; doi:10.1186/s43058-021-00136-7)
Supplement: Supplementary file 2 — Additional file 2. Table A.1 Criteria1 for assigning quantitative ratings to CFIR constructs. [file 43058_2021_136_MOESM2_ESM.docx]

**Table A.1. Criteria^1^ for assigning quantitative ratings to CFIR constructs**

| **Rating** | **Criteria** |
| --- | --- |
| +2 | A construct is a strong positive/facilitating influence if the majority of interviewees provide explicit examples of how the construct manifests itself in a positive way. |
| +1 | A construct is a weak positive/facilitating influence if interviewees make only general statements about the construct manifesting in a positive way, there is a mixed effect of different aspects of the construct but with a general overall positive effect, and/or an indirect inference can be made about the construct having a generally positive influence. |
| 0 (N) | A construct has neutral influence if it is mentioned only generically without evidence of positive or negative influence. |
| 0 (XS) | A construct has strong mixed influence if there are strongly positive and strongly negative influences at different levels in the organization that balance each other out, and/or different aspects of the construct have strong positive influence while others have strong negative influence and overall, the effect is neutral. |
| 0 (XM) | A construct has moderate mixed influence if there are moderate positive and moderate negative influences at different levels in the organization that balance each other out, and/or different aspects of the construct have moderate positive influence while others have moderate negative influence and overall, the effect is neutral. |
| -1 | A construct is a weak negative influence if interviewees make only general statements about the construct manifesting in a negative way, there is a mixed effect of different aspects of the construct but with a general overall negative effect, and/or an indirect inference can be made about the construct having a generally negative influence. |
| -2 | A construct is a strong negative influence if the majority of interviewees give explicit examples of how it impeded implementation efforts or work processes. |

^1^Criteria were adapted from Damschroder and Lowery, *Implementation Science*, 2013.
